# Whole Grain Oats and Barley Products Modify Lipoproteins and Cardiometabolic Risk Markers in Adults: A 6-Week, Double-Blind, Parallel Design, Randomized Controlled Feeding Trial

**DOI:** 10.3390/nu18172873

**Published:** 2026-09-02

**Authors:** Kim S. Stote, David J. Baer, Theresa Henderson, Gloria I. Solano-Aguilar, Anton Terekhov, Bruce R. Hamaker, Janet A. Novotny

**Affiliations:** 1 Beltsville Human Nutrition Research Center, Agricultural Research Service, U.S. Department of Agriculture, Beltsville, MD 20705, USA; david.baer@usda.gov (D.J.B.); theresa.henderson@usda.gov (T.H.); gloria.solano-aguilar@usda.gov (G.I.S.-A.); 2 College of Health Sciences, State University of New York, Empire State University, Saratoga Springs, NY 12866, USA; kim.stote@sunyempire.edu; 3 Whistler Center for Carbohydrate Research, Purdue University, West Lafayette, IN 47907, USA; aterekho@purdue.edu (A.T.); hamakerb@purdue.edu (B.R.H.)

**Keywords:** whole grains, barley, oats, β-glucan, cardiovascular disease, cholesterol, blood pressure, randomized controlled trial, RCT

## Abstract

**Background/Objectives**: Oats and barley are β-glucan-rich grains that may reduce risk factors for cardiovascular disease in adults. This study was conducted to evaluate the effects of products made with either whole grain barley or whole grain oats on CVD risk factors in adults. **Methods**: A 6-week, double-blind, parallel-design, randomized, controlled feeding trial was conducted with 68 adults. Subjects were randomly assigned to either: (1) a control diet containing 0.8 daily servings of whole grain per 2000 kcal, (2) a diet containing 4 daily servings of whole grain oats per 2000 kcal, or (3) a diet containing 4 daily servings of whole grain barley per 2000 kcal. Markers of cardiovascular disease risk were assessed before and after treatment. **Results**: LDL cholesterol concentrations show a trend of statistical significance across diets (*p* = 0.0638), with a lowering of 8.9 mg/dL (barley) and 11.2 mg/dL (oats) compared to the control. Both whole grain diets significantly lowered total VLDL and chylomicron particles (*p* = 0.0272). The oat diet significantly lowered total LDL particles (*p* = 0.0014), small LDL particles (*p* = 0.0008), medium–small LDL particles (*p* = 0.0008), and very small LDL particles (*p* = 0.0009) and increased LDL particle size (*p* = 0.0451). The barley diet significantly lowered diastolic blood pressure (*p* = 0.0388) and vascular cell adhesion molecule concentrations (*p* = 0.0320). **Conclusions**: Diets containing products made from whole grain oats may improve blood lipid profiles, while diets containing products made from whole grain barley may provide additional benefits for blood pressure and inflammation.

## 1. Introduction

A key public health priority in the prevention of cardiovascular disease (CVD) is addressing modifiable risk factors, such as dietary intake [1,2,3]. To that end, increased consumption of whole grains has been associated with a reduced risk of developing CVD [4,5]. The current 2025–2030 Dietary Guidelines for Americans suggest 2–4 servings of whole grains daily. While whole grains are a rich source of dietary fiber, magnesium, potassium, selenium, zinc, iron, iodine, folate, niacin, vitamin E, and phenolic compounds, fiber has been a particular focus of health research, especially with respect to effects on cardiovascular disease, diabetes, and colon cancer [6,7,8].

Dietary fiber is a complex class of carbohydrate polymers that are not digestible in the small intestine, where they can be fermented by microbes. Different chain length and structure affects microbial fermentation in the colon, subsequently resulting in different health effects [9]. β-Glucan is a viscous soluble fiber found in whole grain oats and barley with especially promising effects on blood lipids [10,11]. The US Food and Drug Administration (FDA) has recognized that β-glucan may lower LDL cholesterol, and the US FDA has permitted the use of this health claim in food labeling [12,13]. Systematic reviews and meta-analyses (SRMA) of randomized controlled trials (RCTs) show that barley [14] and oat β-glucan [11,15,16,17] have a lowering effect on LDL cholesterol. Ho et al. [14] reported in a SRMA of 14 RCTs that a median dose of 6.5 mg/day of barley β-glucan for a median duration of 4 weeks significantly lowered LDL cholesterol (−0.25 mmol/L/−9.7 mg/dL); 79% of the 14 RCTs were conducted in those with hypercholesterolemia with a higher proportion of male subjects (male to female ratio 60:40). Another SRMA of 58 RCTs showed that a median dose of 3.5 g/day of oat β-glucan for a median duration of ≥ 3 weeks significantly lowered LDL cholesterol (−0.19 mmol/L/−7.4 mg/dL); 67% of the 58 RCTs were conducted in those with hypercholesterolemia with equal numbers of male and female subjects [16]. Although both oats and barley are rich sources of the soluble fiber β-glucan, they differ in nutrient composition and other properties that may influence physiological effects. As the two richest dietary sources of cereal β-glucan, barley and oat offer particular promise for influencing cardiovascular disease risk factors [11,14].

The processes used to create palatable, shelf-stable food products, such as germination, fermentation, drying, cooking, and extrusion, can alter the physicochemical properties of dietary fiber [18,19], which may influence its physiological effects [20,21]. Although systematic reviews and meta-analyses support the cholesterol-lowering effects of oat and barley β-glucan, relatively few randomized controlled feeding trials have evaluated whole grain oat and barley food *products*, particularly using comprehensive cardiovascular biomarkers beyond conventional blood lipids. Furthermore, comparing whole grain foods rather than isolated β-glucan is important because the food matrix and other grain constituents may influence physiological responses. In addition, very few studies have investigated barley’s effects on cardiometabolic health. Therefore, we conducted a six-week, randomized, double-blind, parallel-design, controlled-feeding study to compare diets containing whole grain oat or whole grain barley cereals and crackers with a refined wheat control diet. We hypothesized that diets containing whole grain oat or barley products would improve traditional and emerging cardiovascular disease risk factors, including lipoprotein particle concentrations, inflammatory markers, and blood pressure, compared with the refined wheat control diet.

## 2. Materials and Methods

### 2.1. Study Subjects

Potential subjects were recruited simultaneously from the Beltsville, MD, USA, area to participate in several upcoming controlled feeding studies at the US Department of Agriculture (USDA), Beltsville Human Nutrition Research Center (BHNRC). Study investigators explained the study requirements, risks, and benefits to volunteers, who then signed a written informed consent form. After providing written informed consent, volunteers completed a health history questionnaire, provided a blood and urine specimen for clinical analysis (profile of biochemical and hematological parameters), and underwent a medical evaluation by a study physician. From this pool of volunteers, the principal investigator invited eligible individuals to participate in the study. All procedures were approved by the MedStar Health Research Institute Institutional Review Board (Hyattsville, MD, USA) and were conducted in accordance with the Declaration of Helsinki. Study subjects received monetary compensation for their participation in the study. The trial was registered at https://clinicaltrials.gov (NCT01293604).

Inclusion criteria were an age of 25–70 years and a Body Mass Index (BMI) between 19 kg/m^2^ and 38 kg/m^2^. Exclusion criteria were as follows: do not regularly consume breakfast or dislike cereal for breakfast; allergy or adverse reaction to study foods; presence of kidney disease, liver disease, gout, untreated or unstable hyperthyroidism, certain cancers, gastrointestinal disease, pancreatic disease, diabetes, other metabolic diseases, or malabsorption syndromes; fasting triglycerides > 300 mg/dL; fasting glucose > 126 mg/dL; blood pressure > 180/100 mmHg; history of bariatric or certain other surgeries related to weight control; history of major surgery within 3 months of enrollment; smokers or other tobacco users (during 6 months prior to the start of the study); antibiotic use during the intervention or for 3 months prior to the intervention period; history of eating disorders or other dietary patterns that are not consistent with the dietary intervention (e.g., high protein diets, vegetarians, very low fat diets); loss of 10% of body weight within the last 6 months; unable or unwilling to give informed consent or communicate with study staff; self-report of alcohol or substance abuse within the past 12 months and/or current acute treatment or rehabilitation program for these problems (long-term participation in Alcoholics Anonymous is not an exclusion); other medical, psychiatric, or behavioral factors that, in the judgment, of the principal investigator may interfere with study participation or the ability to follow the dietary pattern protocol.

### 2.2. Study Design and Diets

The study was a double-blind, parallel design, fully diet-controlled feeding trial with 3 arms implemented for 6 weeks. The randomization plan was generated by the study biostatistician to achieve balance in age, BMI and sex. The biostatistician provided the randomization plan to the study investigators prior to the start of the study. The study was conducted at the USDA BHNRC in Beltsville, MD, USA. Subjects were randomly assigned to 1 of 3 treatment arms, which differed in the type of grain (whole grain oat, whole grain barley, or refined wheat) and the amount of whole grain (mean intake by US adults vs. the recommended daily intake). The treatment arms were (1) a diet containing 0.8 daily servings of whole grain per 2000 kcal and devoid of oats and barley (control arm with the mean whole grain intake by adults in the U.S.), (2) a diet containing 4 daily servings of whole grain oats per 2000 kcal or (3) a diet containing 4 daily servings of whole grain barley per 2000 kcal. The serving of whole grains used in the current study is equivalent to approximately 16 g of whole grains. For the whole grain treatment, 4 servings per 2000 kcal was chosen to be in accord with the Dietary Guidelines for Americans recommendation, and for the control treatment, 0.8 servings were chosen per 2000 kcal to be in accord with the mean intake of whole grains by adults in the U.S. The menus comprised a base diet consumed by all participants as well as grain treatment food products, to produce a controlled 7-day menu cycle served to subjects for 6 weeks. The control, oat and barley food products included cereal bars, coated flakes, coated puffs and crackers (supplied by Kellogg’s, Battle Creek, MI, USA); all other food items consumed were identical among treatment arms. Menus comprised typical American foods and provided approximately 15% of energy from protein, 28% of energy from fat, 9% of energy from saturated fat, and 57% of energy from carbohydrate.

Treatment food items were labeled with color-coded labels, and the items made with different grains were visually identical, so that the study was double-blind. The intervention code was not lifted until all statistical analyses were complete and the data set was locked. Weekday breakfasts and dinners were consumed on-site at BHNRC, and lunches, snacks, and weekend meals were provided for carry-out. During weekdays, the treatment products were supplied at breakfast and dinner so that whole grain food products could be consumed under the supervision of study staff. Study subjects were fed at weight maintenance portions, and body weight was measured before breakfast, Monday through Friday, so that 7–10-day patterns of weight loss or weight gain could be identified and corrected by altering portion sizes for all items to maintain diet composition. Subjects were instructed to eat all foods and only foods provided to them by the USDA BHNRC, apart from water, coffee, tea, and approved diet soda. Coffee and tea intake were limited to 2 cups per day. Consumption of coffee, tea, and diet soda was recorded by study subjects. Alcohol and dietary supplements were prohibited during the study. Diet samples were collected during the controlled feeding phase and subjected to chemical analysis.

### 2.3. Anthropometrics and Blood Pressure

Blood pressure and heart rate data were collected according to a standard protocol, allowing subjects to rest 10 min prior to measurement, and measurements were collected in triplicate, separated by 5 min, using a Welch Allyn Spot Vital Signs instrument (Baxter International, Skaneateles Falls, NY, USA). The participants were instructed to refrain from consuming coffee for at least 30 min prior to the measurement. The means of the three blood pressure and heart rate measurements were used for statistical analysis.

### 2.4. Biological Sample Collection and Analysis

Blood samples were collected after a 12 h fast into serum separator tubes and EDTA-coated vacutainers at the beginning and end of the study treatment period, on 2 different mornings separated by 1 day. Serum and plasma were stored in cryovials at −80 °C until analysis. Serum total cholesterol (Total Chol), HDL cholesterol (HDL Chol), LDL cholesterol (LDL Chol), and triglyceride concentrations were determined by enzymatic procedures using a Vitros Clinical Chemistry Analyzer (Vitros 5, l; QuidelOrtho/Ortho-Clinical Diagnostics, Inc., Rochester, NY, USA). Non-HDL cholesterol (Non-HDL Chol) was calculated as the difference between total cholesterol and HDL cholesterol. The triglyceride-to-HDL cholesterol ratio was calculated by dividing triglyceride concentrations by HDL cholesterol concentrations. Remnant cholesterol was calculated as total cholesterol minus LDL cholesterol minus HDL cholesterol. Comprehensive lipoprotein analysis (LipoProfile Test) was determined by NMR spectroscopy (LipoScience/Labcorp, Raleigh, NC, USA). Serum lipoprotein (a) and apolipoprotein B48 (Apo B48) were measured by immunoassay method (Beckman Coulter Inc., Brea, CA, USA). Serum high sensitivity C-reactive protein (CRP), interleukin-1 beta (IL1-β), interleukin-6 (IL-6), interleukin-8 (IL-8), interleukin-10 (IL-10), interleukin-12 (IL-12), serum amyloid A (SAA), tumor necrosis factor alpha (TNF-α), E-selectin, intercellular adhesion molecule (ICAM) and vascular cell adhesion molecule (VCAM) were analyzed with sandwich-type immunoassay methods using electrochemiluminescence detection (Meso Scale Discovery, Rockville, MD, USA). Factor VII and fibrinogen were measured by automated coagulation/light-scattering assay (Instrumentation Laboratory Company, Bedford, MA, USA) performed on a Coulter ACL coagulation analyzer (Beckman Coulter Inc., Brea, CA, USA).

A dietary fiber analysis of the overall diet, including soluble and insoluble fiber, was conducted according to AOAC 2009.01 and AOAC 2011.25 by Eurofins Nutrition Analysis Center (Wilson, NC, USA). β-Glucan content of the overall diet was analyzed by AOAC 995.16 by Medallion Labs (Minneapolis, MN, USA). Dietary fiber analysis, including β-glucan content and molecular weight, of treatment products was determined as follows: 50 mg of sample was weighed and placed into a 15 mL tube. Next, 2 mL of 95% dimethyl sulfoxide (DMSO) was added, a stir bar was inserted, and the tube was capped. The solution was stirred in a boiling water bath for 2 h and then left stirring overnight at room temperature. Then, 12 mL of 100% ethanol was added, and the mixture was left for 3 h to allow precipitation. The tubes were centrifuged at 5000 rpm for 10 min, and the supernatant was discarded. The tubes were then left open to dry. The above procedure was repeated with the modification as follows: before ethanol was added, the mixture was centrifuged at 5000 rpm for 10 min to remove DMSO-insoluble material. After ethanol precipitation, 4 mg of the dried sample was weighed into a new tube, and 1 mL of HPLC-grade water was added. The solution was stirred in a boiling water bath for 5 h. The resulting solution was filtered through a 0.45 μm nylon filter and analyzed using a Wyatt multi-angle laser light scattering (MALS) system equipped with a refractive index detector. The MALS system was also equipped with two Agilent PL-aquagel-OH 60 and 40 columns connected in series. The mobile phase was water at a flow rate of 0.5 mL/min. The columns were maintained in a 60 °C oven. Astra software v6 (London, UK) was used to calculate molecular weight. Samples were analyzed in duplicate.

### 2.5. Statistical Analysis

To evaluate treatment effects, statistical analyses were performed per protocol using a linear mixed-effects model repeated measures analysis of variance (PROC MIXED procedure in SAS; version 9.4; SAS Institute, North Carolina, USA). The treatment codes were not lifted until the statistical analyses were complete. Covariates of age, baseline (pretreatment), BMI, and sex were retained in all models. The interactions of these covariates with treatment were removed from the model if not significant. If the treatment effects were significant, an all pairwise comparison of treatment groups was conducted, comparing the least square means of the control, barley, and oat groups. Model effects are reported as least squares means ± SE. Data were tested for normality with the Shapiro–Wilk statistic and by inspection of stem–leaf plots and normal probability plots of residuals. In all statistical comparisons, differences with *p* < 0.05 were considered significant. Cohen’s d effect sizes for paired differences between outcome variables were calculated in Excel (Microsoft 365 MSO version 2606, Washington, USA).

The primary outcome variable was LDL cholesterol; therefore, a power calculation was conducted based on data from previous randomized controlled trials from our laboratory. The expected change in LDL cholesterol was 11.8% between the control and treatment diets, based on the mean change observed when study subjects consumed 4 g/d of soluble fiber from barley per 2000 kcal in our laboratory previously and the standard deviation of the difference from previous studies. Based on these assumptions, the sample size needed for a one-way ANOVA, parallel-arm study with 80% power to detect a significant (*p* = 0.05) effect was 19 participants/arm or 19x3 = 54 subjects for the study. Five additional participants were enrolled per arm to account for possible attrition.

## 3. Results

**Figure 1:**
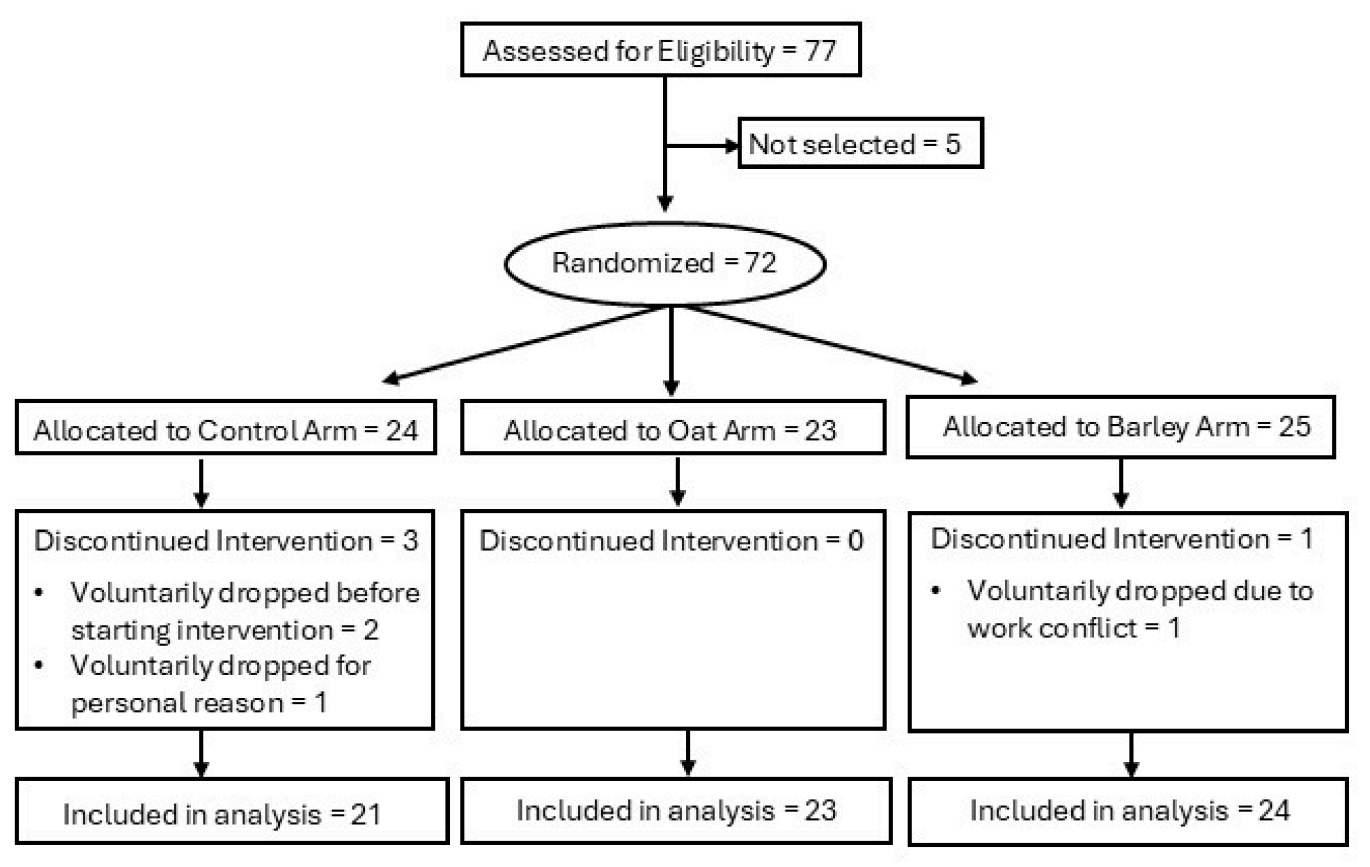
CONSORT diagram for the study trial. CONSORT, Consolidated Standards of Reporting Trials.

Seventy-two study participants were enrolled and randomized from the pool of 77 screened, eligible volunteers who had demonstrated interest in participating in the study. Two volunteers who had been randomized to a treatment decided to withdraw before beginning the intervention. Two additional participants withdrew from the study (one for undisclosed personal reasons and one due to a work conflict). A total of 68 subjects successfully completed the study protocol. Twenty-one subjects completed the control (placebo) arm, 23 subjects completed the whole grain oat arm, and 24 subjects completed the whole grain barley arm (Figure 1). Compliance with the protocol was monitored through daily conversations, supervised meals, regular weight measurements, and data collected on daily questionnaires. No major deviations from the protocol were observed.

The baseline characteristics of the 68 subjects included in the final analysis are presented in Table 1. The subjects included females (*n* = 35) and males (*n* = 33), with an age range of 26–69 years and a BMI range of 20–37 kg/m^2^. The mean ± SE body weight (82.2 ± 1.9 kg vs. 82.2 ± 1.9 kg, respectively; *p* = 0.19) and the mean ± SE BMI (28.5 ± 0.5 kg/m^2^ vs. 28.5 ± 0.5 kg/m^2^, respectively; *p* = 0.13) were not significantly different after 6 weeks of consumption of the dietary treatments. Approximately 16% of subjects (*n* = 10) used antihypertensive medications, with 14.3% (*n* = 3) in the control arm, 17.4% (*n* = 4) in the oat arm, and 16.7% (*n* = 4) in the barley arm. A few subjects, 4.4% (*n* = 3), used lipid-lowering medications, with 9.5% (*n* = 2) in the control arm and 4.2% (*n* = 1) in the barley arm. No medication changes were noted throughout the 6 weeks of the study.

The composition of each diet arm is shown in Table 2. Chemical analyses confirmed that the actual diet composition was similar to the calculated target composition. The control diet was devoid of β-glucan, the oat-containing diet delivered 2.8 g/d β-glucan per 2000 kcal, and the barley-containing diet delivered 5.3 g/d β-glucan per 2000 kcal. The characteristics of the fibers provided by the whole grain treatment foods are shown in Table 3. The barley treatment foods provided more total dietary fiber, insoluble dietary fiber and soluble dietary fiber (both low MW and high MW) than the oat treatment foods. Further, the barley treatment foods contained higher-molecular-weight fibers compared to the oat treatment foods.

Treatment effects on blood lipid concentrations are shown in Table 4 and Table 5. The oat treatment exhibited a moderate lowering effect on LDL cholesterol compared to the control (Cohen’s d = 0.683, 95% CI 0.074–1.292), as well as non-HDL cholesterol compared (Cohen’s d = 0.607, 95% CI 0.001–1.212), and the ratio of total cholesterol to HDL cholesterol (Cohen’s d = 0.703, 95% CI 0.092–1.313). While differences in fasting blood lipids and lipoproteins among the treatment arms did not meet statistical significance at the *p* < 0.05 level, LDL Chol concentrations showed a trend for statistical significance (*p* = 0.0638). LDL Chol for the oat and barley arms were lowered by 11.2 mg/dL and 8.9 mg/dL, respectively, compared to that for the control arm. The Total Chol to HDL Chol ratio also showed a trend for statistical significance (*p* = 0.0584), with the ratio being the lowest after the oat arm. There were no significant interactions of the dietary treatments with age, BMI and sex for lipids, lipoproteins, the total cholesterol:HDL cholesterol ratio, the triglyceride:HDL cholesterol ratio or remnant cholesterol. Non-HDL cholesterol showed a significant interaction between the dietary treatments and sex (*p* = 0.0064).

Treatment effects on lipoprotein particle concentrations are shown in Table 6 and Table 7. Total VLDL and chylomicron particles were significantly lower after the whole grain oat and barley treatments (*p* = 0.0272) compared to the control diet. Total VLDL and chylomicron particles showed significant interactions for age (*p* = 0.0028) and BMI (*p* = 0.0081). Small VLDL particles were significantly (*p* = 0.0411) lowered for the oat and barley diets when compared to the control diet. The effect sizes for VLDL and chylomicron particles were small, with 95% CI including 0. Total LDL particles (*p* = 0.0014), small LDL particles (*p* = 0.0008), medium–small LDL particles (*p* = 0.0008) and very small LDL particles (*p* = 0.0009) were significantly lowered in the oat arm compared to control. LDL size was significantly (*p* = 0.0451) affected by dietary treatment, with the LDL size being higher after the oat diet. The lowering effect of oats was large for total LDL particles (Cohen’s d = 0.992, 95% CI 0.393–1.620 for control comparison; Cohen’s d = 0.975, 95% CI 0.368–1.581 for barley comparison), medium–small LDL particles (Cohen’s d = 1.071, 95% CI 0.437–1.706 for control comparison; Cohen’s d = 0.972 95% CI 0.366–1.578 for barley comparison), small LDL particles (Cohen’s d = 0.995, 95% CI 0.366–1.624 for control comparison; Cohen’s d = 1.065, 95% CI 0.452–1.678 for barley comparison), and very small LDL particles (Cohen’s d = 0.961, 95% CI 0.335–1.587 for control comparison; Cohen’s d = 1.071, 95% CI 0.458–1.684 for barley comparison). The oat treatment effect on LDL size compared to the barley treatment was also large (Cohen’s d = 0.825, 95% CI 0.228–1.422). There were no significant interactions of the dietary treatment with age, BMI and sex. However, large HDL particles showed a significant interaction with age (*p* = 0.0423).

The treatment effects on markers of inflammation and hemostasis are shown in Table 8 and Table 9. The adhesion molecule VCAM was significantly lowered (*p* = 0.0320) after the barley diet compared to control, while differences in other biomarkers of inflammation and hemostasis did not meet statistical significance. The barley treatment exhibited a large lowering effect on VCAM (Cohen’s d = 0.805, 95% CI 0.195–1.415). The oat treatment exhibited a moderate effect in increasing IL-10 (Cohen’s d = 0.625, 95% CI 0.018–1.231). IL-1β showed a significant interaction with sex (*p* = 0.0272). IL-8 and ICAM showed a significant interaction with BMI (*p* = 0.0017 and *p* = 0.0298, respectively). Fibrinogen showed a significant interaction with age (*p* = 0.0347).

The effects of the whole grain treatments on blood pressure are shown in Table 10 and Table 11. Diastolic blood pressure was significantly lowered after the barley diet (−3.0 mm Hg, *p* = 0.0388) compared to the control, while systolic blood pressure was not affected. Mean arterial pressure (MAP) showed marginal statistical significance (*p* = 0.062), with MAP being the lowest after the barley diet.

## 4. Discussion

CVD remains a significant public health challenge, being the number one killer worldwide, with dietary modification serving as a cornerstone for prevention strategies [1,2,3]. The current study contributes valuable evidence to support the health-promoting properties of whole grain food products, particularly those incorporating oat or barley, for the generally healthy adult population. The trend for a reduction in LDL cholesterol concentrations with both the whole grain barley- and whole grain oat-containing diets supports prior evidence from systematic reviews and meta-analyses [11,14,15,16,17]. While the reductions in LDL cholesterol of −0.29 mmol/L (−11.2 mg/dL) for oats and −0.23 mmol/L (−8.9 mg/dL) for barley did not reach statistical significance (*p* = 0.0638), the magnitude and direction of change are consistent with previous evidence supporting the LDL cholesterol-lowering effects of cereal β-glucan. Further, the observed effect sizes support important health benefits of consuming oat-containing food products. These findings should be interpreted cautiously given the sample size and warrant confirmation in larger studies. Research shows that each 1 mmol/L reduction in LDL cholesterol (~38.7 mg/dL) is associated with a 25% decrease in major cardiovascular events; therefore, risk reduction with an LDL cholesterol reduction of 10 mg/dL would be 5–6% [22].

Few human RCTs have investigated advanced lipoprotein particle analyses in studies of whole grain oat [23] or barley [24,25]. In the current study, subjects had a mean baseline LDL particle concentration exceeding the desirable range of less than 1000 nmol/L. The lipoprotein particle analysis demonstrated a significant lowering of total VLDL and chylomicron particles for whole grain oat and barley diets compared to the control diet. Notably, only the oat-containing diet significantly lowered total LDL particles, small LDL particles, and improved LDL size, all of which are crucial in reducing atherogenic risk. LDL cholesterol serves as a surrogate marker of LDL particle concentrations [26]. However, baseline data showed that subjects’ mean LDL particles were above the recommended range. A given mass of cholesterol transported by smaller, more numerous particles creates a more atherogenic environment compared to the same mass carried by fewer, larger LDL particles. Consequently, those with a high number of small, dense LDL particles are at the greatest risk for atherosclerosis. LDL particle number may be a stronger predictor of CVD risk than LDL cholesterol [27].

The primary mechanism proposed for the cholesterol-lowering effects of soluble fibers such as β-glucan, is their ability to trap bile acids and cholesterol, reducing their absorption and reabsorption. This decreased absorption leads to lower levels of cholesterol and bile acids in hepatocytes, which in turn stimulates the upregulation of hepatic LDL receptors. These receptors remove apolipoprotein B-containing lipoproteins, including LDL particles, from the bloodstream, thereby lowering circulating LDL cholesterol levels [28,29]. While the fermentability of dietary fibers may contribute to their cholesterol-lowering effects, this is thought to play a more secondary role, and the evidence supporting this is inconsistent [29,30].

Research on the relationship between whole grain consumption and inflammatory response has yielded mixed results in humans [31,32]. This inconsistency can be attributed to several factors, including differences in study design, the types of whole grains studied, subject characteristics, and biomarkers of inflammation measured [33]. Also, few human RCTs have evaluated barley and oat consumption and hemostasis [34,35,36,37]. In the current study, the adhesion molecule VCAM was significantly lowered after the barley diet compared to the control diet. VCAM plays a key role in regulating inflammation-related vascular adhesion and the trans-endothelial migration of leukocytes, including macrophages and T cells. Some evidence links VCAM to the progression of several immunological disorders, such as rheumatoid arthritis and cancer [38]. To our knowledge, there are no human RCTs that have evaluated adhesion molecules and barley intake. This finding suggests that barley may possess anti-inflammatory properties that warrant further investigation [39]. However, other inflammatory and hemostatic biomarkers showed no significant changes, underscoring the need for more sensitive and longer-term assessments.

The whole grain barley diet lowered diastolic blood pressure, with reductions of approximately 3.0 mm Hg compared to the control diet in normotensive adults. While systolic blood pressure and mean arterial pressure showed marginal or no significance, the diastolic blood pressure lowering is meaningful as it reflects improvements in vascular resistance, an essential component of cardiovascular health. A 3.0 mm Hg decrease in diastolic blood pressure is linked to an approximately 6% reduction in stroke risk and a 4% reduction in CVD risk [40]. There is limited data on barley consumption and blood pressure, though Behall et al. found a 2.9 mm Hg decrease in diastolic blood pressure in hypercholesterolemic adults after consuming a barley dietary pattern for 5 weeks in a highly controlled feeding study, which aligns with the current study [25]. In contrast, Smith et al. found no effect on either systolic or diastolic blood pressure after 6 weeks with low molecular weight or high molecular weight barley in hypercholesterolemic adults following a free-living diet [41], though the subjects were not on a controlled diet. The current study’s result also aligns with an SRMA suggesting that whole grain consumption, particularly oat β-glucan, may have beneficial effects on vascular function in adults at low risk for CVD, though these SRMAs did not include any barley RCTs [42]. Thus, barley’s role in reducing LDL cholesterol uptake may improve the elasticity of blood vessel walls, which decreases vascular resistance, thereby lowering blood pressure [43].

Although both oat and barley diets contained β-glucan-rich whole grain products and improved several cardiovascular risk markers, differences in physiological responses were observed. The oat-containing diet resulted in significant improvements in the LDL particle number, small LDL particles, and LDL particle size, whereas the barley-containing diet produced significant reductions in VCAM and diastolic blood pressure. These differences may reflect variation in grain composition, β-glucan concentration, molecular characteristics, and other properties that influence lipid metabolism and vascular function. While the oat products were lower in fiber and β-glucan, the smaller particle size of the oat β-glucan may have provided greater solubility and functional viscosity in the intestinal tract, improving the mechanistic health benefits. In addition, oats are rich in avenanthramides, which may have provided additional beneficial effects.

A key strength of this study is its controlled feeding design, which minimizes confounding factors such as dietary variability, and the high level of investigator-subject interaction and study supervision strengthens compliance with the prescribed study diets. By using whole grain products in practical forms (e.g., bars, cereals and crackers), the study also reflects real-world applicability. Furthermore, the analysis of multiple CVD biomarkers, including lipoprotein particles and inflammatory markers, provides a comprehensive evaluation of the cardiovascular benefits of whole grain consumption. A limitation of this study may have been the sample size, as the results for LDL cholesterol just missed statistical significance at the *p* < 0.05 level. In addition, the reported results for the secondary outcome variables were not the primary outcome variable, so caution is warranted in their interpretation. Examining numerous secondary outcomes increases the risk of a Type I error (false positive) [44]. Nevertheless, several secondary outcomes showed statistically significant results with *p* < 0.05. It is, therefore, unlikely that all observed significant findings are solely due to Type I error [45].

## 5. Conclusions

The current study provides additional evidence that whole grain food products containing oats and barley may favorably influence selected cardiovascular disease risk markers, including LDL cholesterol, atherogenic lipoprotein particles, inflammation-related biomarkers, and blood pressure in adults at low risk for CVD. These findings are consistent with current dietary recommendations to increase whole grain intake as part of a heart-healthy dietary pattern. Incorporating a variety of whole grains, including oats and barley, may provide complementary health benefits; however, larger and longer-term randomized controlled trials are needed to confirm these effects and determine their impact on cardiovascular disease risk.

## Figures and Tables

**Table 1:** Baseline characteristics of subjects who completed the intervention.

Characteristic	Treatment Arm		
	Control (n = 21)	Oat (n = 23)	Barley (n = 24)
Age (y)	53.2 ± 2.4	54.0 ± 1.6	49.9 ± 2.6
Sex (n)	M = 11, F = 10	M = 11, F = 12	M = 11, F = 13
Height (cm)	170.5 ± 1.9	168.2 ± 1.9	169.9 ± 1.8
Weight (kg)	82.0 ± 3.4	78.8 ± 3.2	85.6 ± 3.2
Body mass index (kg/m^2^)	28.1 ± 0.9	27.7 ± 0.8	29.6 ± 0.9
Total cholesterol (mg/dL)	196.7 ± 8.0	193.0 ± 6.7	201.7 ± 6.2
LDL cholesterol (mg/dL)	120.8 ± 7.6	116.6 ± 7.2	130.2 ± 5.8
HDL cholesterol (mg/dL)	52.8 ± 3.0	56.3 ± 3.2	50.9 ± 2.0
Non-HDL cholesterol (mg/dL)	143.9 ± 8.5	136.8 ± 6.8	150.8 ± 6.0
Triglycerides (mg/dL)	114.3 ± 14.2	85.2 ± 7.5	108.4 ± 9.9
Systolic blood pressure (mmHg)	115.5 ± 2.7	119.3 ± 3.4	109.5 ± 1.9
Diastolic blood pressure (mmHg)	70.5 ± 1.8	73.2 ± 2.0	68.2 ± 1.4

Values are presented as mean ± SE for continuous variables.

**Table 2:** Energy, macronutrient, and grain content of study diets.

Component	Treatment Arm		
	Control	Oat	Barley
Protein (% of energy)	14.2	14.8	14.9
Fat (% of energy)	27.6	28.2	28.1
Carbohydrate (% of energy)	58.2	57.0	56.9
Soluble dietary fiber (g/d) ^1^	4.1	6.6	10.9
Insoluble dietary fiber (g/d) ^1^	30.7	32.2	33.7
Total dietary fiber (g/d) ^1^	34.8	38.8	44.6
β-Glucan (g/d) ^1^	0	2.8	5.3
Whole grains (servings/d)^1^	0.8	4.2	4.2
Whole grains (g/d) ^1^	13	67	67

^1^ Values are presented per 2000 kcal.

**Table 3:** Characteristics of dietary fiber provided by treatment foods.

Component	Treatment	
	Oat	Barley
β-glucan (g/d) ^1^	2.8	5.3
Low MW soluble dietary fiber (g/d) ^1^	0.9	1.5
High MW soluble dietary fiber (g/d) ^1^	3.1	7.0
Soluble dietary fiber (g/d) ^1^	4.1	8.5
Insoluble dietary fiber (g/d) ^1^	4.1	5.8
Total dietary fiber (g/d) ^1^	8.2	14.3
MW of fiber particles (Da)	2.5–7.5 × 10^6^	6.1–11.9 × 10^6^

^1^ Values are presented per 2000 kcal.

**Table 4:** Fasting biomarkers of lipids and lipoproteins before and after the dietary intervention ^1^.

	Pre-Intervention ^2^			Post-Intervention ^3^			
Variable	Control	Oat	Barley	Control	Oat	Barley	*p* ^4^
Total Chol (mg/dL)	196.7 ± 8.0	193 ± 6.7	201.7 ± 6.2	195.8 ± 3.9	185.7 ± 3.8	187.2 ± 3.7	0.1554
LDL Chol (mg/dL)	120.8 ± 7.6	116.6 ± 7.2	130.2 ± 5.8	124.6 ± 3.6	113.4 ± 3.4	115.7 ± 3.4	0.0638
HDL Chol (mg/dL)	52.8 ± 3.0	56.3 ± 3.2	50.9 ± 2.0	51.0 ± 1.1	51.8 ± 1.1	49.9 ± 1.1	0.4845
Non-HDL Chol (mg/dL)	143.9 ± 8.5	136.8 ± 6.8	150.8 ± 6.0	144.4 ± 3.6	134.3 ± 3.5	137.0 ± 3.4	0.1127
Triglyceride (mg/dL)	114.3 ± 14.2	85.2 ± 7.5	108.35 ± 9.9	107.2 ± 6.0	111.0 ± 5.8	107.3 ± 5.7	0.8726
Total Chol:HDL Chol	4.0 ± 0.3	3.7 ± 0.3	4.1 ± 0.2	4.1 ± 0.1	3.77 ± 0.1	3.9 ± 0.1	0.0584
Triglyceride:HDL Chol	2.5 ± 0.5	1.8 ± 0.3	2.5 ± 0.3	2.4 ± 0.2	2.5 ± 0.1	2.4 ±0.1	0.7039
Remnant Chol (mg/dL)	23.1 ± 2.9	20.2 ± 1.6	20.6 ± 1.6	20.2 ± 1.2	20.7 ± 1.2	21.2 ± 1.2	0.8386
Lipoprotein (a) (mg/dL)	20.6 ± 3.0	22.6 ± 6.0	39.06 ± 6.5	29.9 ± 1.2	31.1 ± 1.1	29.7 ± 1.2	0.6297
Apo B48 (mg/dL)	0.109 ± 0.019	0.666 ± 0.006	0.686 ± 0.008	0.0833 ± 0.008	0.075 ± 0.007	0.082 ± 0.007	0.7448

^1^ Control arm n = 21, Oat arm n = 23, Barley arm n = 24. ^2^ Values are arithmetic means ± SEs from samples collected before dietary intervention. ^3^ Values are least-squares means ± SEs from an ANOVA linear mixed model that included covariates of baseline values, age, BMI, and sex. ^4^ Values are for control vs. treatment at 6 weeks.

**Table 5:** Effect sizes for blood lipids.

			95% Confidence Interval	
Variable	Comparison	Cohen’s d Effect Size	Lower	Upper
Total Chol	Control vs. Oat	0.559	−0.044	1.163
	Control vs. Barley	0.478	−0.117	1.072
	Oat vs. Barley	0.083	−0.490	0.655
LDL Chol	Control vs. Oat	0.683	0.074	1.292
	Control vs. Barley	0.537	−0.060	1.133
	Oat vs. Barley	0.140	−0.433	0.712
HDL Chol	Control vs. Oat	0.155	−0.438	0.747
	Control vs. Barley	0.210	−0.377	0.798
	Oat vs. Barley	0.356	−0.220	0.933
Non-HDL Chol	Control vs. Oat	0.607	0.001	1.212
	Control vs. Barley	0.446	−0.147	1.039
	Oat vs. Barley	0.161	−0.411	0.734
Triglyceride (TG)	Control vs. Oat	0.137	−0.455	0.730
	Control vs. Barley	0.004	−0.582	0.589
	Oat vs. Barley	0.133	−0.440	0.705
Tot Chol:HDL Chol	Control vs. Oat	0.703	0.092	1.313
	Control vs. Barley	0.421	−0.172	1.013
	Oat vs. Barley	0.268	−0.306	0.843
TG:HDL Chol	Control vs. Oat	0.232	−0.361	0.826
	Control vs. Barley	0.030	−0.556	0.616
	Oat vs. Barley	0.200	−0.373	0.773
Remnant Chol	Control vs. Oat	0.088	−0.504	0.680
	Control vs. Barley	0.180	−0.407	0.766
	Oat vs. Barley	0.092	−0.480	0.665
Lp(a)	Control vs. Oat	0.223	−0.371	0.816
	Control vs. Barley	0.035	−0.551	0.621
	Oat vs. Barley	0.250	−0.324	0.825
Apo B48	Control vs. Oat	0.237	−0.357	0.830
	Control vs. Barley	0.037	−0.549	0.622
	Oat vs. Barley	0.206	−0.367	0.780

**Table 6:** Lipoprotein particle concentrations and size before and after the dietary intervention ^1^.

	Pre-Intervention ^2^			Post-Intervention ^3^			
Variable	Control	Oat	Barley	Control	Oat	Barley	*p* ^4^
VLDL and chylomicron (CM) particles							
VLDL and CM particles, total (nmol/L)	67.4 ± 7.3	49.4 ± 4.6	64.0 ± 5.4	66.1 ± 4.3 ^a^	59.55 ± 4.4 ^b^	57.67 ± 4.9 ^b^	0.0272
Large VLDL and CM particles (nmol/L)	3.0 ± 0.9	1.03 ± 0.4	2.3 ± 0.6	2.6 ± 0.5	2.4 ± 0.4	2.1 ± 0.4	0.7275
Medium VLDL particles (nmol/L)	27.8 ± 5.4	19.3 ± 2.9	25.9 ± 4.8	30.3 ± 3.1	31.8 ± 3.0	26.9 ± 3.0	0.5140
Small VLDL particles (nmol/L)	36.7 ± 3.3	29.0 ± 2.6	35.8 ± 2.7	33.6 ± 2.7 ^a^	27.1 ± 2.7 ^b^	27.6 ± 3.0 ^b^	0.0411
VLDL size (nm)	48.6 ± 1.3	49.1 ± 1.3	50.3 ± 1.4	50.37 ± 1.1	51.35 ± 1.1	51.78 ± 1.1	0.6413
LDL particles							
LDL particles, total (nmol/L)	1293.0 ± 93.3	1236.7 ± 94.5	1346.2 ± 67.3	1359.3 ± 48.7 ^a^	1137.3 ± 46.8 ^b^	1358.2 ± 46.7 ^a^	0.0014
IDL particles (nmol/L)	34.0 ± 9.3	26.4 ± 7.2	26.0 ± 6.6	39.9 ± 5.9	23.6 ± 5.7	30.8 ± 5.7	0.1416
Large LDL particles (nmol/L)	417.0 ± 50.2	521.7 ± 45.0	486.5 ± 42.7	422.1 ± 27.1	430.7 ± 25.6	386.2 ± 25.4	0.4443
Medium–small LDL particles (nmol/L)	174.0 ± 20.2	143.7 ± 21.9	174.6 ± 16.7	194.2 ± 11.0 ^a^	140.5 ± 10.4 ^b^	189.5 ± 10.4 ^a^	0.0008
Small LDL particles (nmol/L)	842.1 ± 101.4	688.6 ± 112.6	833.8 ± 77.9	914.6 ± 52.2 ^a^	677.3 ± 49.6 ^b^	933.4 ± 49.6 ^a^	0.0008
Very small LDL particles (nmol/L)	668.0 ± 81.4	544.9 ± 90.9	659.1 ± 61.3	720.9 ± 42.0 ^a^	536.5 ± 39.9 ^b^	743.7 ± 39.9 ^a^	0.0009
LDL size (nm)	20.9 ± 0.2	21.4 ± 0.2	21.0 ± 0.1	20.9 ± 0.1 ^a^	21.1 ± 0.1 ^b^	20.7 ± 0.1 ^a^	0.0451
HDL particles							
HDL particles, total (μmol/L)	34.1 ± 0.7	33.2 ± 1.4	34.3 ± 0.8	32.5 ± 0.6	31.8 ± 0.6	32.7 ± 0.6	0.4272
Large HDL particles (μmol/L)	8.5 ± 1.1	10.0 ± 0.9	7.9 ± 0.7	9.0 ± 0.3	8.3 ± 0.3	8.7 ± 0.3	0.3555
Medium HDL particles (μmol/L)	4.7 ± 0.09	3.7 ± 0.6	3.9 ± 0.6	2.8 ± 0.5	2.5 ± 0.5	3.5 ± 0.5	0.4145
Small HDL particles (μmol/L)	20.9 ± 1.2	19.5 ± 0.7	22.5 ± 0.8	21.2 ± 0.7	20.4 ± 0.7	21.0 ± 0.7	0.6685
HDL size (nm)	9.0 ± 0.1	9.2 ± 0.1	9.0 ± 0.1	9.0 ± 0.0	9.0 ± 0.0	9.0 ± 0.0	0.2024

^1^ Control arm n = 21, oat arm n = 23, barley arm n = 24. ^2^ Values are arithmetic means ± SEs from samples collected before dietary intervention. ^3^ Values are least-squares means ± SEs from an ANOVA linear mixed model that included covariates of baseline values, age, BMI, and sex. ^4^ Values are for control vs. treatment at 6 weeks. Different superscript letters in a row indicate statistically significant differences, *p* < 0.05.

**Table 7:** Effect sizes for lipoprotein particle size and concentration of different particle sizes.

			95% Confidence Interval	
Variable	Comparison	Cohen’s d Effect Size	Lower	Upper
VLDL/CM total	Control vs. Oat	0.320	−0.275	0.916
	Control vs. Barley	0.381	−0.210	0.972
	Oat vs. Barley	0.083	−0.489	0.655
Large VLDL/CM	Control vs. Oat	0.095	−0.497	0.687
	Control vs. Barley	0.236	−0.352	0.824
	Oat vs. Barley	0.155	−0.418	0.727
Medium VLDL	Control vs. Oat	0.105	−0.487	0.697
	Control vs. Barley	0.235	−0.353	0.823
	Oat vs. Barley	0.337	−0.239	0.913
Small VLDL	Control vs. Oat	0.513	−0.089	1.114
	Control vs. Barley	0.439	−0.154	1.032
	Oat vs. Barley	0.036	−0.536	0.608
VLDL size	Control vs. Oat	0.190	−0.403	0.783
	Control vs. Barley	0.270	−0.319	0.858
	Oat vs. Barley	0.081	−0.492	0.653
LDL total	Control vs. Oat	0.992	0.363	1.620
	Control vs. Barley	0.005	−0.581	0.591
	Oat vs. Barley	0.975	0.368	1.581
IDL	Control vs. Oat	0.599	−0.006	1.205
	Control vs. Barley	0.331	−0.259	0.921
	Oat vs. Barley	0.261	−0.314	0.835
Large LDL	Control vs. Oat	0.070	−0.522	0.661
	Control vs. Barley	0.289	−0.300	0.878
	Oat vs. Barley	0.360	−0.217	0.937
Medium–small LDL	Control vs. Oat	1.071	0.437	1.706
	Control vs. Barley	0.093	−0.493	0.679
	Oat vs. Barley	0.972	0.366	1.578
Small LDL	Control vs. Oat	0.995	0.366	1.624
	Control vs. Barley	0.078	−0.508	0.664
	Oat vs. Barley	1.065	0.452	1.678
Very small LDL	Control vs. Oat	0.961	0.335	1.587
	Control vs. Barley	0.117	−0.469	0.704
	Oat vs. Barley	1.071	0.458	1.684
LDL size	Control vs. Oat	0.426	−0.173	1.024
	Control vs. Barley	0.421	−0.172	1.013
	Oat vs. Barley	0.825	0.228	1.422
HDL total	Control vs. Oat	0.248	−0.345	0.842
	Control vs. Barley	0.070	−0.516	0.656
	Oat vs. Barley	0.309	−0.266	0.885
Large HDL	Control vs. Oat	0.497	−0.104	1.098
	Control vs. Barley	0.210	−0.377	0.798
	Oat vs. Barley	0.275	−0.300	0.850
Medium HDL	Control vs. Oat	0.128	−0.464	0.720
	Control vs. Barley	0.294	−0.294	0.883
	Oat vs. Barley	0.412	−0.166	0.991
Small HDL	Control vs. Oat	0.243	−0.350	0.837
	Control vs. Barley	0.060	−0.526	0.646
	Oat vs. Barley	0.177	−0.396	0.750
HDL size	Control vs. Oat	0.000	−0.592	0.592
	Control vs. Barley	0.000	−0.586	0.586
	Oat vs. Barley	0.000	−0.572	0.572

**Table 8:** Fasting biomarkers of inflammation and hemostasis before and after the dietary intervention ^1^.

	Pre-Intervention ^2^			Post-Intervention ^3^			
Variable	Control	Oat	Barley	Control	Oat	Barley	*p* ^4^
CRP (mg/dL)	3.0 ± 0.7	1.4 ± 0.3	3.3 ± 0.8	3.2 ± 0.6	2.4 ± 0.6	2.2 ± 0.6	0.5005
IL-1β (pg/dL)	0.7 ± 0.2	0.8 ± 0.2	0.5 ± 0.1	0.7 ± 0.1	0.6 ± 0.1	0.7 ± 0.1	0.1978
IL-6 (pg/dL)	2.1 ± 0.5	1.4 ± 0.1	2.3 ± 0.7	1.5 ± 0.1	1.5 ± 0.1	1.6 ± 0.1	0.6451
IL-8 (pg/dL)	10.0 ± 0.8	10.4 ± 0.9	9.4 ± 0.7	9.6 ± 0.6	9.7 ± 0.5	8.6 ± 0.5	0.2669
IL-10 (pg/dL)	10.4 ± 4.1	6.2 ± 1.8	14.2 ± 5.8	7.8 ± 3.6	18.2 ± 3.5	10.6 ± 3.4	0.1174
IL-12 (pg/dL)	10.2 ± 2.9	8.8 ± 3.6	18.3 ± 7.9	9.6 ± 10.7	10.9 ± 10.3	34.4 ± 10.3	0.1871
TNF-α (pg/mL)	14.4 ± 5.2	9.1 ± 0.5	10.3 ± 1.0	10.5 ± 0.4	10.0 ± 0.4	10.1 ± 0.4	0.5684
E-selectin (ng/mL)	15.0 ± 1.7	16.6 ± 1.5	13.7 ± 1.4	14.9 ± 0.6	15.1 ± 0.6	15.0 ± 0.6	0.9699
SAA (μg/mL)	3.32 ± 0.58	2.14 ± 0.30	3.77 ± 1.06	3.47 ± 0.41	3.33 ± 0.39	3.53 ± 0.39	0.9358
ICAM (ng/mL)	260.9 ± 13.3	245.5 ± 9.4	251.9 ± 11.8	267.9 ± 7.2	257.1 ± 6.9	249.2 ± 6.9	0.1922
VCAM (mg/mL)	349.2 ± 21.6	360.8 ± 15.6	372.4 ± 17.0	413.8 ± 8.4 ^a^	402.3 ± 8.0 ^b^	382.5 ± 8.0 ^a,b^	0.0320
Factor VII (%)	105.5 ± 4.6	98.1 ± 4.2	107.2 ± 3.8	98.6 ± 1.7	97.9 ± 1.7	100.5 ± 1.6	0.5171
Fibrinogen (mg/dL)	355.6 ± 14.4	338.1 ± 9.6	400.9 ± 14.4	374.2 ± 7.7	378.4 ± 7.7	368.0 ± 7.7	0.6608

^1^ Control arm n = 21, oat arm n = 23, barley arm n = 24. ^2^ Values are arithmetic means ± SEs from samples collected before dietary intervention. ^3^ Values are least-squares means ± SEs from an ANOVA linear mixed model that included covariates of baseline values, age, BMI, and sex. ^4^ Values are for control vs. treatment at 6 weeks. Different superscript letters in a row indicate statistically significant differences, *p* < 0.05.

**Table 9:** Effect sizes for biomarkers of inflammation and hemostasis.

			95% Confidence Interval	
Variable	Comparison	Cohen’s d Effect Size	Lower	Upper
CRP	Control vs. Oat	0.284	−0.311	0.879
	Control vs. Barley	0.351	−0.240	0.941
	Oat vs. Barley	0.069	−0.503	0.641
IL-1β	Control vs. Oat	0.213	−0.380	0.806
	Control vs. Barley	0.000	−0.586	0.586
	Oat vs. Barley	0.206	−0.367	0.780
IL-6	Control vs. Oat	0.000	−0.592	0.592
	Control vs. Barley	0.210	−0.377	0.798
	Oat vs. Barley	0.206	−0.367	0.780
IL-8	Control vs. Oat	0.039	−0.553	0.631
	Control vs. Barley	0.386	−0.206	0.977
	Oat vs. Barley	0.454	−0.126	1.033
IL-10	Control vs. Oat	0.625	0.018	1.231
	Control vs. Barley	0.169	−0.418	0.756
	Oat vs. Barley	0.455	−0.125	1.034
IL-12	Control vs. Oat	0.026	−0.565	0.618
	Control vs. Barley	0.498	−0.097	1.093
	Oat vs. Barley	0.471	−0.110	1.051
TNF-α	Control vs. Oat	0.266	−0.328	0.861
	Control vs. Barley	0.210	−0.377	0.798
	Oat vs. Barley	0.052	−0.520	0.624
E-selectin	Control vs. Oat	0.071	−0.521	0.663
	Control vs. Barley	0.035	−0.551	0.621
	Oat vs. Barley	0.034	−0.538	0.606
SAA	Control vs. Oat	0.075	−0.517	0.666
	Control vs. Barley	0.032	−0.554	0.617
	Oat vs. Barley	0.106	−0.467	0.678
ICAM	Control vs. Oat	0.327	−0.269	0.923
	Control vs. Barley	0.559	−0.038	1.157
	Oat vs. Barley	0.236	−0.338	0.810
VCAM	Control vs. Oat	0.299	−0.296	0.894
	Control vs. Barley	0.805	0.195	1.415
	Oat vs. Barley	0.510	−0.071	1.092
Factor VII	Control vs. Oat	0.088	−0.504	0.680
	Control vs. Barley	0.243	−0.345	0.831
	Oat vs. Barley	0.325	−0.251	0.901
Fibrinogen	Control vs. Oat	0.116	−0.476	0.708
	Control vs. Barley	0.169	−0.417	0.756
	Oat vs. Barley	0.279	−0.296	0.853

**Table 10:** Blood pressure before and after the dietary intervention ^1^.

	Pre-Intervention ^2^			Post-Intervention ^3^			
Variable	Control	Oat	Barley	Control	Oat	Barley	*p* ^4^
Systolic BP (mm Hg)	115.8 ± 3.3	117.7 ± 3.3	106.1 ± 1.6	115.1 ± 1.5	113.6 ± 1.5	110.9 ± 1.5	0.1562
Diastolic BP (mm Hg)	71.0 ± 1.7	73.1 ± 2.0	66.3 ± 1.3	71.2 ± 1.7 ^a^	71.1 ± 0.9 ^a^	68.2 ± 0.9 ^b^	0.0388
Mean Arterial BP (mm Hg)	85.9 ± 2.1	88.0 ± 2.3	79.6 ± 1.3	85.7 ± 2.1	85.2 ± 1.0	82.4 ± 1.0	0.062

^1^ Control arm n = 21, oat arm n = 23, barley arm n = 24. ^2^ Values are arithmetic mean SEs from samples collected before dietary intervention. ^3^ Values are least-squares mean SEs from an ANOVA linear mixed model that included covariates of baseline values, age, BMI, and sex. ^4^ Values are for control vs. treatment at 6 weeks. Different superscript letters in a row indicate statistically significant differences, *p* < 0.05.

**Table 11:** Effect sizes for blood pressure.

			95% Confidence Interval	
Variable	Comparison	Cohen’s d Effect Size	Lower	Upper
Systolic BP	Control vs. Oat	0.213	−0.380	0.806
	Control vs. Barley	0.589	−0.010	1.188
	Oat vs. Barley	0.371	−0.206	0.948
Diastolic BP	Control vs. Oat	0.016	−0.576	0.608
	Control vs. Barley	0.483	−0.112	1.077
	Oat vs. Barley	0.665	0.076	1.253
Mean Arterial BP	Control vs. Oat	0.067	−0.525	0.658
	Control vs. Barley	0.441	−0.152	1.034
	Oat vs. Barley	0.577	−0.007	1.162

## Data Availability

The original data presented in the study are openly available in Ag Data Commons at https://agdatacommons.nal.usda.gov/.

## Abbreviations

The following abbreviations are used in this manuscript:

ANOVA	Analysis of variance
AOAC	Association of Official Analytical Collaboration
BHNRC	Beltsville Human Nutrition Research Center
BMI	Body mass index
Chol	Cholesterol
CRP	C-reactive protein
CVD	Cardiovascular disease
DGA	Dietary Guidelines for Americans
DMSO	Dimethyl sulfoxide
FDA	Food and Drug Administration
HDL	High-density lipoproteins
HPLC	High-performance liquid chromatography
ICAM	Intracellular adhesion molecule
IDL	Intermediate density lipoproteins
IL-1β	Interleukin 1 beta
IL-6	Interleukin 6
IL-8	Interleukin 8
IL-10	Interleukin 10
IL-12	Interleukin 12
LDL	Low-density lipoproteins
MALS	Multi-angle light scattering
RCT	Randomized controlled trial
SAA	Serum amyloid A
SE	Standard error
SRMA	Systematic review and meta-analysis
TNF-α	Tumor necrosis factor alpha
US	United States
USDA	United States Department of Agriculture
VCAM	Vascular cell adhesion molecule
VLDL	Very low-density lipoproteins
